# Using Electrospinning-Based Carbon Nanofiber Webs for Methanol Crossover Control in Passive Direct Methanol Fuel Cells

**DOI:** 10.3390/ma11010071

**Published:** 2018-01-04

**Authors:** Wei Yuan, Guoyun Fang, Zongtao Li, Yonghui Chen, Yong Tang

**Affiliations:** School of Mechanical and Automotive Engineering, South China University of Technology, Guangzhou 510640, China; megyfang@mail.scut.edu.cn (G.F.); meztli@scut.edu.cn (Z.L.); meyhchen@mail.scut.edu.cn (Y.C.); ytang@scut.edu.cn (Y.T.)

**Keywords:** direct methanol fuel cell, passive, methanol crossover, carbon nanofiber webs, electrospinning

## Abstract

Methanol crossover (MCO) significantly affects the performance of a direct methanol fuel cell (DMFC). In order to reduce its effect, this study presents in-house carbon nanofiber webs (CNWs) used as a porous methanol barrier for MCO control in a passive DMFC. The CNW is made from polyacrylonitrile (PAN) by using electrospinning and heat treatment. The impacts of PAN concentration and carbonizing temperature on the material properties are considered. The concentration of PAN has a great effect on the micro structures of the CNWs since a higher concentration of PAN leads to a larger nanofiber diameter and lower porosity. A higher carbonizing temperature helps promote the sample conductivity. The use of CNWs has twofold effects on the cell performance. It helps significantly enhance the cell performance, especially at a low methanol concentration due to its balanced effect on reactant and product management. There is an increase in peak power density of up to 53.54% when the CNW is used, in contrast with the conventional DMFC at 2 mol/L. The dynamic and constant-load performances of the fuel cell based on CNWs are also investigated in this work.

## 1. Introduction

The rapid development of direct methanol fuel cell (DMFC) technology accelerates the wide application of low-power portable devices due to its advantages such as low-temperature operation, high power density and safe fuel storage [1]. In this field, a special type of DMFC based on the passive methanol/oxygen feeding mode is attracting increasing attention. Different from the active system with pumps, blowers, heat exchangers and so on, a passive DMFC eliminates accessional apparatus, depending on spontaneous mass and heat transfer mechanisms. Therefore, it has a compact structure and low cost. Nonetheless, the passive DMFC faces several obstacles before it is put into practical use and commercialization. It has been widely known that methanol crossover (MCO) happens unavoidably in the Nafion-based membrane when methanol arrives at the anode [2,3]. The permeated methanol directly reacts with the oxygen on the cathode side and thereby forms a mixed potential pulling down the voltage output. It also causes severe fuel waste and catalyst poisoning.

In order to improve the cell performance and fuel efficiency, researchers have made many efforts to develop high efficient catalysts to promote the methanol oxidation reaction at the anode while increasing the methanol tolerance of cathodic catalyst. Some studies concentrate on how to reduce MCO from the perspective of structural design and component optimization. In this regard, an innovative method is to introduce a mass-transfer-controlling medium at the anode to hold back methanol transport. Li et al. [4] added a porous PTFE sheet between the anode chamber and current collector. Their results showed that it was useful to suppress the MCO and improve the fuel utilization at a methanol concentration of 16 mol/L, but the use of a high methanol concentration reduced water flux and lead to membrane dehydration on the anode side. Nakagawa et al. [5] made a comparison among several types of carbon materials used to support the anodic membrane electrode assembly (MEA). They concluded that the mass transfer resistance of the anode diffusion medium had a great effect on the MCO flux and validated the effectiveness of employing the porous plate to reduce methanol waste. Shaffer and Wang [6] introduced an anode transport barrier to resist the methanol and water crossover between the anode catalyst layer and fuel chamber. This method was able to reduce both the methanol and water fluxes to guarantee regular operation at a relatively high methanol concentration. Pan [7] adopted a four-layer Nafion membrane as the buffer zone in front of current collectors, reducing the MCO-related voltage loss as well as increasing the limited current density. Kim et al. [8] employed hydrogel as the diffusion controlling agent to adjust the methanol permeation rate and successfully promoted the cell performance at an optimal fuel concentration of 6 mol/L. Wu et al. [9] used a porous titanium plate as an integrated methanol barrier/current collector which yielded the maximum power density with neat methanol supplement. Yuan et al. [10] fabricated a porous metal fiber sintered plate to alleviate the MCO. 

As is well-known, carbon-based materials such as carbon paper and carbon cloth have been widely used as mass transfer media and reaction substrates in fuel cells. The carbon fibers can construct a porous permeable structure for reactant/product diffusion. In recent years, graphene has attracted much attention due to its excellent electrical conductivity and extra high specific surface area. An example was reported by Xu et al. [11] who tried to combine the graphene with a porous metal to fabricate nanocomposite materials to improve the cell performance. However, graphene also has evident disadvantages such as high-cost and relatively poor machinability.

As aforementioned, many studies demonstrate that using a permeable but resistant material is an effective way to regulate the MCO. However, in the case of a passive DMFC, its mass transfer mechanism is spontaneous, so the process of reactant delivery and product removal is very slow. Under this condition, the use of a controllable porous methanol barrier must help maintain sufficient methanol supply to avoid concentration polarization and concurrently reduce MCO, thereby enhancing the voltage output [12]. This requires that the involved mass transfer medium must be flexible to design and easy to make. More importantly, the material must have controllable structural parameters and functional characteristics in terms of mass transfer. With this purpose, this work developed a functional material based on carbon nanofiber webs (CNWs) to serve as a methanol barrier to mitigate MCO and promote the cell performance. A series of structural parameters and related influence mechanisms were also demonstrated in this paper.

## 2. Results and Discussion

### 2.1. Characterization of the CNWs

**Figure 1:**
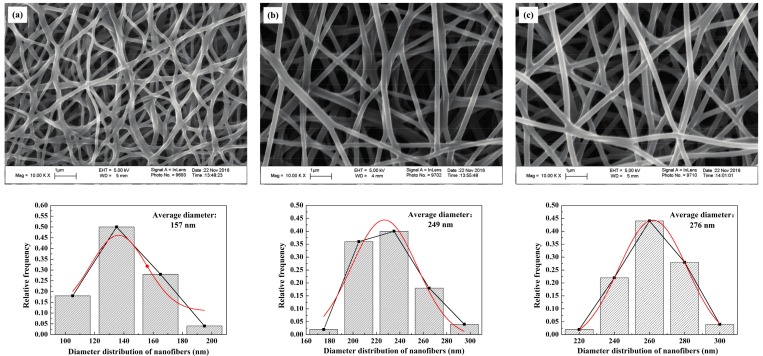
SEM images of the self-developed carbon nanofiber webs (CNWs) in the magnification of 10,000 and diameter distribution of nanofibers with different polyacrylonitrile (PAN) concentrations: (**a**) *C*_L_; (**b**) *C*_M_; (**c**) *C*_H_.

**Figure 2:**
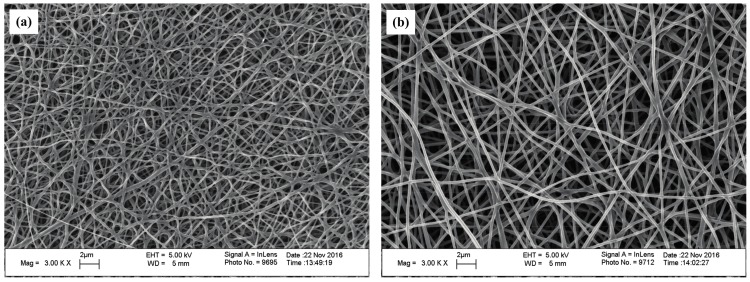
SEM images of the self-developed CNWs in the magnification of 3000: (**a**) *C*_L_; (**b**) *C*_H_.

The SEM images of the self-developed CNWs and diameter distribution of nanofibers with different PAN concentrations are shown in Figure 1. The nanofibers in this material have a regular and flexuous fibrous morphology, whose diameter becomes larger from 157, 249 to 276 nm as the PAN concentration increases from 10.96% (lower concentration, *C*_L_), 13.70% (moderate concentration, *C*_M_) to 16.44% (higher concentration, *C*_H_). Sintering bonds are formed at the junctions of nanofibers, especially at a lower concentration of PAN, i.e., *C*_L_. This phenomenon is greatly relieved as the PAN concentration increases. The final fiber diameter is subject to the diameter of the micro droplet separated when spinning. The solution concentration relates to the viscosity, electrical conductivity and surface tension of the samples [13]. Using a more concentrated solution makes it more difficult to separate the smaller droplets so as to increase the fiber diameter. Table 1 lists some physical properties of the CNWs, including the penetrating time of a 4 μL droplet of 4 mol/L methanol solution. On the basis of the wettability test, the self-made CNWs have an unstable hydrophilicity. The indicator will gradually penetrate into the samples as time progresses. Table 1 indicates that a higher concentration relates to a shorter penetrating time. Especially when the *C*_L_ is used, it yields a result several times larger than that of the *C*_M_ and *C*_H_. This is because a smaller nanofiber diameter directly results in a more compact structure. Moreover, the sintering bonds tend to lower the porosity of the CNWs and thus reduce the permeability of the material to some extent. As shown in Figure 2, it is evident that the *C*_L_ has a smaller diameter, lower porosity and higher compactness. The thickness of the sample increases from 42.61 to 116.49 μm with the increase in solution concentration, due to a larger diameter of fibers and more layers of stacked fibers. Besides, as shown in Table 1, the electrical resistance of the CNW sample is influenced by the status of fiber distribution to a large extent since a higher compactness level and more sintering pastes will lead to a lower electrical resistance, which is helpful to improve the performance of current transfer. Furthermore, these CNW samples are quite light weight with a level of approximately 0.1 g.

**Figure 3:**
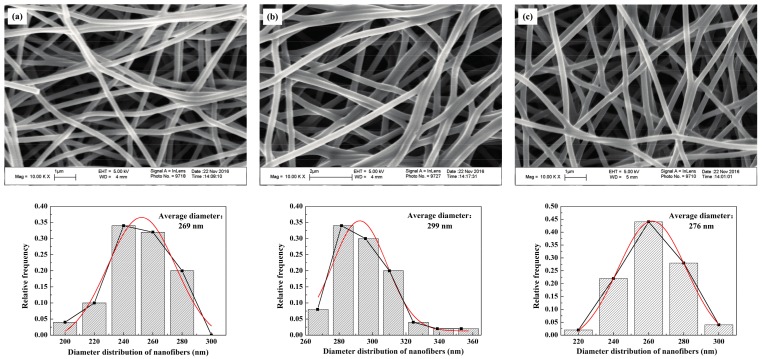
SEM images of the self-developed CNWs in the magnification of 10,000 and diameter distribution of nanofibers with different carbonizing temperatures: (**a**) *T*_L_; (**b**) *T*_M_; (**c**) *T*_H_.

The SEM images of the CNWs and diameter distribution of nanofibers in correspondence with different carbonizing temperatures are shown in Figure 3. Generally, there is no apparent difference in the micro structures between the samples. The values of pore size and diameter are insensitive to the change of temperature. Besides, the levels of sintering bonds and porosities are similar to each other when different temperatures are used. In contrast, the electrical resistance of each sample decreases dramatically with the increase in carbonizing temperature, as the resistivity of the *T*_L_, *T*_M_ and *T*_H_ are 123.33 × 10^−3^, 6.15 × 10^−3^ and 1.92 × 10^−3^ Ω·m, respectively. This is because the hydrogen, oxygen and nitrogen can react under a high-temperature condition and fade away so that the proportion of carbon will increase substantially after 800 °C to cause a sharp drop in its proportion [14]. This explains why the use of *T*_L_ yields a higher electrical resistance in the presence of a higher nitrogen ratio. *T*_H_ reacts at a relatively high carbonizing temperature which brings about a lower electrical resistance. In the temperature range from 800 to 1250 °C, the increase of carbonizing temperature is beneficial to the improvement of conductivity.

**Figure 4:**
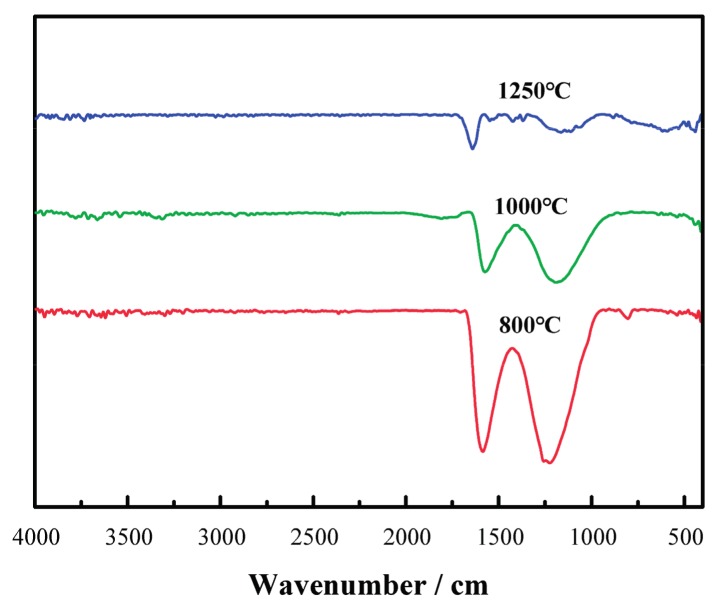
Infrared Spectroscopy of the samples at different carbonizing temperatures.

As shown in Figure 4, with the increase in carbonizing temperature, the absorption peaks of -COO- band characteristics at 1220 cm^−1^ and -C=N- features in the band at 1590 cm^−1^ decrease significantly. This indicates the increased growth in the degrees of cyclization, deoxygenization and denitrification reaction to reach a higher level of carbonization of the samples.

### 2.2. Effects of the PAN Concentration on the Performance of DMFCs

**Figure 5:**
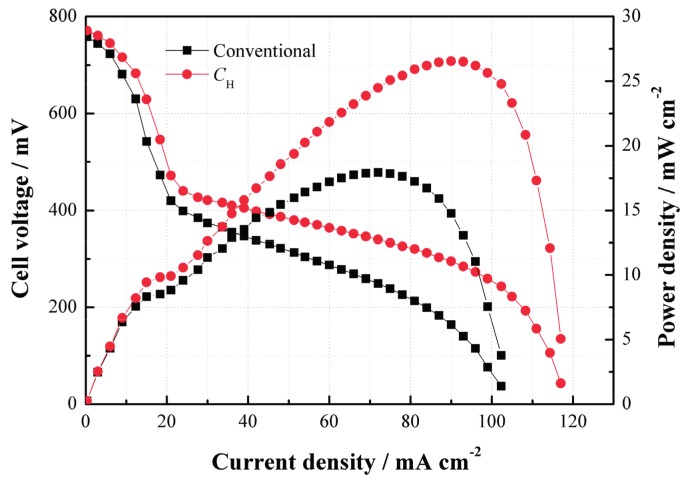
Performance comparisons between the passive direct methanol fuel cells (DMFCs) with and without *C*_H_ at 2 mol/L.

In order to evaluate the effect of the CNWs on the performance of a DMFC, the cell performance curves were measured when 2 mol/L methanol was used. Figure 5 compares the performances of the cells with *C*_H_ and the conventional structure. As can be seen, the power density of the DMFC with *C*_H_ is much higher than that without *C*_H_ at 2 mol/L. Such performance improvement benefits from the use of *C*_H_ which helps increase the methanol mass transfer resistance and thus limit the MCO degree as well as improve the performance due to its internal porous structure.

**Figure 6:**
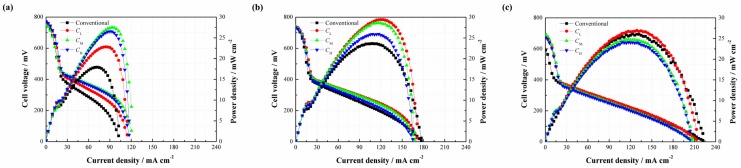
Performance comparisons between the passive DMFCs without and with *C*_L_, *C*_M_ and *C*_H_ at different methanol concentrations: (**a**) 2 mol/L; (**b**) 4 mol/L; (**c**) 6 mol/L.

Since the CNWs are proved to benefit the performance of the DMFC, it is necessary to investigate the effect of the PAN concentration on the performance of the DMFC (see Figure 6). As shown in Figure 6a, three different types of CNW-based DMFCs yield a higher cell performance than the conventional setup at 2 mol/L. It is understandable that the CNWs play the role of methanol barrier to directly increase its mass transfer resistance. Besides, the hydrophilcity of the CNWs can also facilitate the water transport but indirectly inhibit the methanol transfer [15]. These two aspects are both beneficial to alleviate the MCO. The peak power densities of *C*_L_, *C*_M_ and *C*_H_ are 22.85, 27.53 and 26.55 mW/cm^2^, whereas the conventional DMFC yields 17.93 mW/cm^2^. In contrast, the samples based on *C*_L_, *C*_M_ and *C*_H_ improve by 27.44%, 53.54% and 48.08%, respectively. This is because the sintering degree of *C*_L_ is high and the diameter of its nanofiber is smaller. As a result, the methanol transfer resistance becomes higher, so the MCO is greatly restricted by its compact structure. However, in the meantime, mass transfer becomes more difficult. In this case, the methanol supplement may not be sufficient in the fuel cell reaction at the anode, especially when a low methanol concentration is used. This means that the cell also possibly suffers from fuel starvation. Conversely, the sintering degree of *C*_H_ is much lower and its fiber diameter is larger than *C*_L_. In other words, the methanol transfer resistance can be much lower in this situation, which helps accelerate the chemical reaction, facilitate the cell performance, but possibly brings about a higher MCO degree. The different behaviors verify that the mass transfer mechanism is a critical issue for a passive DMFC. The use of *C*_M_ produces the highest peak power density due to its moderate sintering degree and suitable size of the carbon nanofiber. An appropriate mass transfer resistance is able to not only meet the demand of methanol supplement but also alleviate MCO effectively. In this study, it is recommended to use the CNWs, especially the pattern of *C*_M_, to improve the cell performance at a lower methanol concentration.

When the methanol concentration rises to 4 mol/L, it can be found that three different types of DMFCs with the CNWs still achieve higher peak power densities than that of the conventional DMFC. However, the performance gap becomes smaller, as shown in Figure 6b. On the other hand, the cell performances from high to low are *C*_L_, *C*_M_ and *C*_H_. This phenomenon can be explained as follows. When the concentration reaches 4 mol/L, the methanol transfer to the anode catalyst layer becomes easier. This is also the case for the process of MCO. The negative effect of methanol starvation is greatly reduced while MCO is more and more severe. In the case of *C*_L_, it has a relatively high mass transfer resistance to inhibit the methanol penetration from the anode to the cathode, which helps limit the MCO as much as possible and thus yields a better performance.

However, as shown in Figure 6c, it is observed that the performance of *C*_L_ is slightly higher than that of the conventional one as the fuel concentration rises to 6 mol/L, while *C*_M_ and *C*_H_ exhibit even worse performances. This is because the internal reaction in the fuel cell becomes intense, consuming more methanol and generating more CO_2_. The unmodified structure of the DMFC makes the CO_2_ bubbles escape more easily from the diffusion layer to the outside compartment. However, the use of CNWs is not beneficial to CO_2_ removal. On one hand, it increases the mass transfer resistance of bubble extrusion from the gas diffusion layer into the flow field. On the other hand, the hydrophilic surface favors the formation of CO_2_ bubbles of smaller size and more uniform distribution [16,17]. As time progresses, small and separate bubbles, attached onto the inside wall of the CNWs, increasingly gather together, which will raise the internal pressure and block the feeding path of methanol fuel. Moreover, the water produced at the cathode should be removed as quickly as possible, otherwise it will hold back oxygen intake. Under the effect of the CNWs, the process of water backflow from the cathode to the anode becomes difficult due to the increase of anode pressure. Though the existence of CNWs greatly restrains the MCO, it also hinders the removal of the product. This section demonstrates that the cell performance is determined by diverse factors under different conditions.

### 2.3. Effects of the Carbonizing Temperature on the Performance of DMFCs

**Figure 7:**
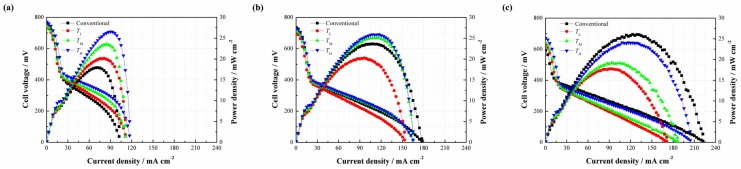
Performance comparisons between the passive DMFCs without and with *T*_L_, *T*_M_ and *T*_H_ at different methanol concentrations: (**a**) 2 mol/L; (**b**) 4 mol/L; (**c**) 6 mol/L.

This section focuses on how the cell performance varies with the change of carbonizing temperature. Figure 7 illustrates the effects of different carbonizing temperatures on the cell performance. As shown in Figure 7a, three different types of DMFCs with the CNWs yield better cell performances than that of the conventional DMFC without additional CNW at a methanol concentration of 2 mol/L. This result shows a consistent trend with the cases related to different PAN concentrations, and further verifies that the CNWs are helpful to improve the performance of DMFCs. The peak power densities of TL, TM and TH are 20.17, 23.52 and 26.55 mW/cm^2^, corresponding to a 27.44%, 53.54% and 48.08% improvement respectively, compared with the conventional DMFC. There is no apparent difference in the micro structure, including nanofiber diameter, porosity and the thickness among three patterns of the CNWs with different carbonizing temperatures shown in Table 2. This means that the mass transfer resistances of these samples are nearly equal to each other. However, the conductivity of each sample increases rapidly as the temperature increases. This phenomenon highlights why the TL performs much worse than the other two samples due to its high electrical resistance in the presence of a higher nitrogen ratio. The TH reacts at a relatively high carbonizing temperature which leads to a lower resistance and yields the best cell performance.

As shown in Figure 7b, the performances of *T*_M_ and *T*_H_ are slightly higher than that of the conventional DMFC at 4 mol/L, while *T*_L_ shows a lower performance due to the large difference of electrical resistance among the samples. From Figure 7c, it is observed that the positive effect of the CNW even disappears as the concentration of the methanol solution rises to 6 mol/L. This phenomenon is like the effect of PAN concentration at the same methanol concentration, which can be explained by the CO_2_ trapping behaviors.

### 2.4. Dynamic Characteristics

**Figure 8:**
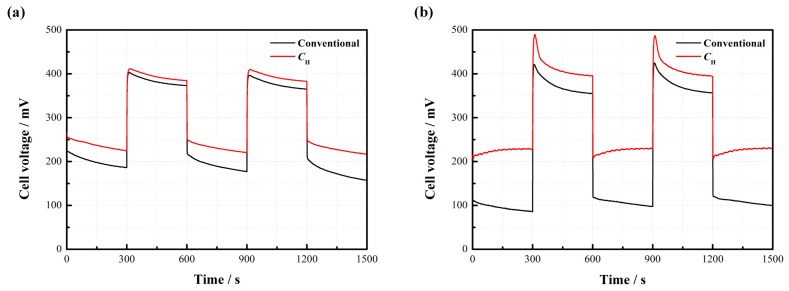
Dynamic characteristics of the passive DMFCs with and without *C*_H_: (**a**) repeated change from 20 to 50 mA/cm^2^ at 2 mol/L; (**b**) repeated change from 20 to 80 mA/cm^2^ at 4 mol/L.

Figure 8 displays the dynamic characteristics of the passive DMFCs with and without *C*_H_. Figure 8a indicates that the cell voltage instantly changes with the repeated change of current density from 20 to 50 mA/cm^2^ at 2 mol/L. Both kinds of cells maintain a relatively stable output in each steady state. It is observed that the cell with *C*_H_ works more stably and has less voltage loss compared to the conventional pattern without *C*_H_. Figure 8b further suggests that the voltage of the DMFC with *C*_H_ responds more rapidly to the change of current density. When the current density sharply drops from 80 to 20 mA/cm^2^, an apparent voltage overshoot happens. After this, the cell voltage gradually drops down until reaching a new stable level. This phenomenon can be attributed to the fact that an abrupt decline of the current load reduces the demand for methanol supply, so the MCO may inevitably build up, thus inducing more voltage losses. Once a new concentration balance between the anode and the cathode is formed, the cell performance can be stabilized. The overshoot degree is closely related to the fluctuation amplitude of the current density [18]. In addition, it is noted that the average cell voltage output with *C*_H_ is much higher than the conventional one, especially during the high-current operation.

### 2.5. Stability Evaluation

**Figure 9:**
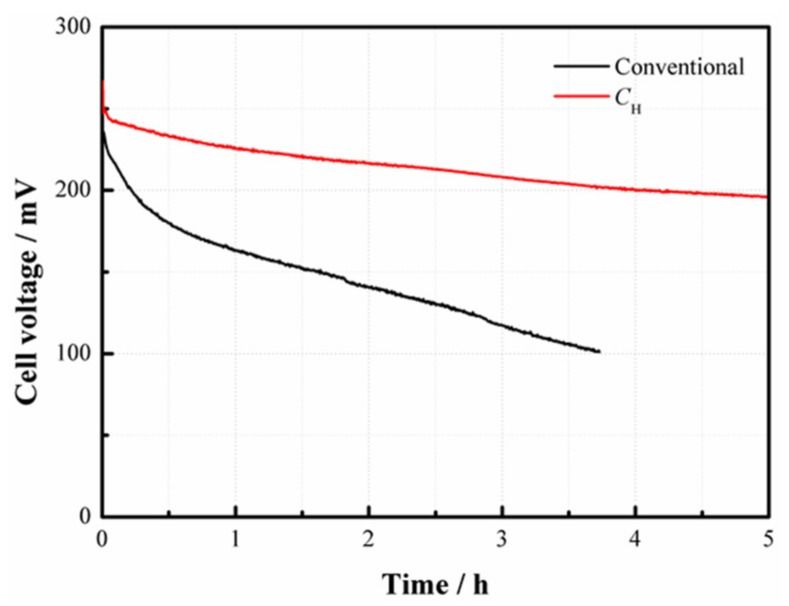
Stability test of the passive DMFCs with and without *C*_H_ at a constant current density of 100 mA/cm^2^ at 4 mol/L.

To investigate the operational stability of the fuel cell, two patterns with and without *C*_H_ are also tested at a constant current density of 100 mA/cm^2^ at 4 mol/L, as depicted in Figure 9. Results demonstrate that the conventional DMFC has an evident voltage decline with an amplitude of about 50% after 3 h. This can be ascribed to the fact that the cell performance is gradually degraded due to the continuous effect of MCO. However, the cell with *C*_H_ still maintains a relatively high voltage output after 5 h operation, simply yielding a decrease of about 10%. This is because the use of CNWs enables the cell to operate at a higher methanol concentration and also a higher current density. As a result, it is more able to meet the demand for methanol fuel in the high-current regions at a reasonable degree of MCO.

## 3. Materials and Methods 

### 3.1. Fabrication of the Carbon Nanofiber Webs (CNWs)

The CNWs were made by electrospinning and heat treatment. The precursor polyacrylonitrile (PAN, Mw = 150,000, Shanghai Macklin Biochemical Co., Ltd., Shanghai, China) was mixed with solvent *N*,*N*-dimethyl formamide (DMF, Shanghai Richjoint Co., Ltd., Shanghai, China) at an appropriate ratio. According to our previous study, in order to achieve a better spinability of nanofibers, three different ratios were applied to inspect the influence of the PAN concentration as shown in Table 2. The mixed solution was treated in a magnetic stirrer at 60 °C for about 3 h until the PAN was dispersed uniformly. Three kinds of polymer solutions with different concentrations were electrospun to form a syringe tip on a piece of copper sheet with a size of 60 mm × 60 mm. Below are the conditions for electrospinning: a feeding rate of 0.4 mL/h polymer solution, a supply voltage of 15 kV, a tip-to-sheet distance of 14 cm, and the use of a 22 G syringe needle with an inner diameter of 0.4 mm. After electrospinning, the PAN-based nanofiber webs were heated at a heating rate of 10 °C/min and stabilized at 270 °C in air for 2 h in a muffle furnace to form pre-stabilized nanofibers. There were two treatment steps including the pre-carbonizing (usually at 400–600 °C) and carbonizing (usually at 600–1300 °C), respectively. Firstly, the pre-stabilized nanofibers were heated at 5 °C/min and stabilized with flowing nitrogen gas at a rate of 50 mL/min for 30 min in a tube furnace at 600 °C. After this, the heating temperature was increased to a higher level for 40 min to complete the final carbonizing process. Three different carbonizing temperatures were evaluated in this experiment to investigate its effect on the properties of the CNWs. 

The nanostructures of the CNWs were characterized by field emission scanning electron microscopy (FESEM, Zeiss^®^, LEO1530VP, Oberkochen, Germany). The surface contact angle was measured using a commercial device (Powereach^®^, JC2000D, Shanghai, China). A 4 μL droplet of 4 mol/L methanol (Guangzhou Chemical Reagent, Inc., Guangzhou, China) solution was used as the indicator. The composition of the CNWs was analyzed by Fourier transform infrared spectroscopy (FTIR, Bruker^®^, Karlsruhe, Germany, VERTEX 33, transmission mode: KBr method).

### 3.2. Preparation of the Passive Air-Breathing Direct Methanol Fuel Cells

Both the anode chamber and cathode window plate were made of polymethylmethacrylate (PMMA). A fuel reservoir with a volume of 11 mL was fabricated in the anode chamber. Two through holes were made on the top of the reservoir for fuel injection and gas venting. Both the anode and cathode current collectors were made of 1 mm-thick SUS316L stainless steel plate with perforated hole-arrays distributed within the active area. In this study, the anode current collector has 144 (12 × 12) holes (d = 1.5 mm) with a lower open ratio of 28.3% while the cathode current collector has 49 (7 × 7) holes (d = 3 mm) with a higher open ratio of 38.5%. Two pairs of rubber gaskets and PTFE films were used to prevent leakage between each component. The as-prepared CNW was located between the MEA and anode current collector. The properties of MEA were as follows:

- Membrane: Nafion^®^ 117 (Dupont, Inc., Wilmington, DE, USA);
- Catalyst loading on the cathode side: 2 mg/cm^2^ Pt (Johnson Matthey, Inc., London, UK);
- Catalyst loading on the anode side: 4 mg/cm^2^ Pt-Ru (Johnson Matthey, Inc., London, UK);
- Cathode gas diffusion layer: wet-proofed carbon paper (Toray, Inc., Tokyo, Japan);
- Anode gas diffusion layer: untreated carbon paper (Toray, Inc., Tokyo, Japan);
- Active area: 30 mm × 30 mm.

### 3.3. Testing Setup and Experimental Strategy

Before each performance testing run, the MEA must be activated at a constant load for 12 h to obtain credible data under a stable operating condition. An electronic load (GE/FC2) was used to provide discharging function and data acquisition. When each test ended, the anode chamber was cleaned with de-ionized water to eliminate the effects of unconsumed methanol in the reservoir on the following test. The ambient temperature and relative humidity were maintained respectively at 25 °C and 85% throughout the whole experiment. The results were characterized by using current vs. voltage (I–V) and current vs. power (I–P) curves.

## 4. Conclusions

This study reports on the preparation and application of self-made CNWs based on the methods of electrospinning and heat treatment. This functional material is used to control the MCO in a passive air-breathing DMFC. The characterization results indicate that using a higher concentration of PAN relates to a larger nanofiber diameter and lower porosity. Additionally, a higher carbonizing temperature generally leads to a higher conductivity. The DMFC equipped with CNWs has a higher cell performance than that with a traditional structure, especially at a lower methanol concentration, e.g., 2 mol/L. The reason for such performance improvement can be attributed to its balanced effect between the fuel supplement and MCO control. In the case of a higher methanol concentration, the positive effect of the CNWs becomes weaker. This is because the cell inevitably encounters great difficulty in CO_2_ gas removal at the anode since the CNWs increase the mass transfer resistance. The dynamic tests also verify the feasibility of using CNWs for improving the performance of a passive DMFC.

## Figures and Tables

**Table 1:** Sample patterns with different penetrating times of methanol solution and resistivity.

Sample Pattern	Penetrating Time of 4 M Methanol (s)	Average Thickness (μm)	Resistivity (×10^−3^ Ω·m)
*C*_L_	16	42.61	1.10
*C*_M_	2	73.01	1.38
*C*_H_	1.8	116.49	1.92

**Table 2:** Sample patterns with different PAN concentrations and carbonizing temperatures.

Sample Pattern	PAN:DMF (g:mL)	Carbonizing Temperature (°C)
*C*_L_	0.8:7.3	1250
*C*_M_	1.0:7.3	1250
*C*_H_	1.2:7.3	1250
*T*_L_	1.2:7.3	800
*T*_M_	1.2:7.3	1000
*T*_H_	1.2:7.3	1250
